# Brazilein Suppresses Inflammation through Inactivation of IRAK4-NF-κB Pathway in LPS-Induced Raw264.7 Macrophage Cells

**DOI:** 10.3390/ijms161126048

**Published:** 2015-11-18

**Authors:** Kui-Jin Kim, Kye-Yoon Yoon, Hyung-Sun Yoon, Sei-Ryang Oh, Boo-Yong Lee

**Affiliations:** 1 Department of Food Science and Biotechnology, CHA University, 335 Pangyo-ro, Bundang-gu, Seongnam-si, Gyeonggi-do 463-300, Korea; Kuijin.Kim@gmail.com (K.-J.K.); beautyygy@naver.com (K.-Y.Y.); 2 Department of Biomedical Laboratory Science, Soon Chun Hyang University, Asan, Chungnam 336-745, Korea; hyoun@suv.ac.kr; 3 Natural Medicine Research Center, Korea Research Institute of Bioscience and Biotechnology, 30 Yeongudanji-ro, Ochang-eup, Cheongwon-gu, Cheongju-si, Chungbuk 363-883, Korea; seiryang@kribb.re.kr

**Keywords:** brazilein, inflammation, functional food, health benefits, NF-κB, toll-like receptors, MAPK, IRAK4

## Abstract

The medicinal herbal plant has been commonly used for prevention and intervention of disease and health promotions worldwide. Brazilein is a bioactive compound extracted from *Caesalpinia sappan* Linn. Several studies have showed that brazilein exhibited the immune suppressive effect and anti-oxidative function. However, the molecular targets of brazilein for inflammation prevention have remained elusive. Here, we investigated the mechanism underlying the inhibitory effect of brazilein on LPS-induced inflammatory response in Raw264.7 macrophage cells. We demonstrated that brazilein decreased the expression of IRAK4 protein led to the suppression of MAPK signaling and IKKβ, and subsequent inactivation of NF-κB and COX2 thus promoting the expression of the downstream target pro-inflammatory cytokines such as IL-1β, MCP-1, MIP-2, and IL-6 in LPS-induced Raw264.7 macrophage cells. Moreover, we observed that brazilein reduced the production of nitrite compared to the control in LPS-induced Raw264.7. Thus, we suggest that brazilein might be a useful bioactive compound for the prevention of IRAK-NF-κB pathway associated chronic diseases.

## 1. Introduction

The immune system is required for host defense and recognizes a variety of pathogen-associated molecular patterns (PAMPs) of invading microbial pathogens. Exposure of immune cells, including macrophages, to specific agonist activates complex signaling cascades that rapidly trigger production of chemokines and pro-inflammatory cytokines, notably macrophage inflammatory protein-2 (MIP2), interleukin-6 (IL-6), tumor necrosis factor-alpha (TNFα), nitric oxide (NO), inducible nitric oxide synthase (iNOS), and cyclooxygenase-2 (COX2), plays a crucial role of initiating inflammatory response [1,2,3].

Toll-like receptor (TLR) plays an important role in the immune response against bacterial or viral infections, and progression of an adaptive immunity [4,5,6]. Lipopolysaccharide (LPS) from Gram-negative bacteria interacts with TLR4 to induce systemic inflammation [7]. TLR trigger the activation of the myeloid differential factor (MyD88) dependent signaling pathway and toll-interleukin-1 receptor domain-containing adapter inducing interferon-β (TRIF) dependent signaling pathway [8,9]. MyD88 activates the IL-1 receptor-associated kinase-4 (IRAK4) and tumor necrosis factor receptor-activated factor 6 (TRAF6) [10]. These adaptor proteins lead to activation of transforming growth factor-beta-activated kinase 1 (TAK1) [11], which then activate the downstream IκB kinase-α (IKKα) and IKKβ and mitogen-activated protein (MAP) 3-kinase pathways (extracellular signal-regulated protein kinase (ERK), c-Jun terminal kinase (JNK), and p38 MAPK) [12]. Phosphorylation of IKKα/β leads to activation of nuclear factor-kappa B (NF-κB) via degradation of IκBα, and the subsequent nuclear translocation of NF-κB [13,14].

In addition, activation of MAPK pathways also leads to the activation of activator protein 1 (AP-1) [15]. In contrast, TRIF induces the downstream activation of Tank-binding kinase-1 (TBK1) and IKKε in the TRIF-dependent pathway [16]. Transcriptional activation of interferon-regulatory factor 3 (IRF3) by these kinases regulates the expression of interferon-β (IFN-β) [17]. The activation of these signaling pathways causes an excessive innate immune response, which can lead to chronic inflammatory diseases, such as cerebral ischemic injury [18], rheumatoid arthritis [19], and atherosclerosis [20]. Thus, identification of new phytochemicals that regulate TLR signaling will facilitate the development of an anti-inflammatory agent for prevention and treatment of inflammation-associated disease.

Brazilein is found in the dried heartwood of *Caesalpinia sappan Linn*., which has been used in traditional herbal medicine in Asia-Pacific region. Previous studies have shown that the main compounds in *Caesalpinia sappan*, including brazilein and brazilin, suppress LPS-induced expression of iNOS and the production of NO in various immune cell lines [21,22]. Moreover, brazilein had a protective effect in a cerebral ischemia/reperfusion model, mediated by its anti-inflammatory activity [23], and induced apoptosis of mice spleen lymphocytes by blocking immunocompetence [24]. Zhong *et al.* reported that brazilein causes the apoptosis through the suppression of survivin protein in HepG2 cells [25]. However, the molecular mechanisms underlying the anti-inflammatory effects of brazilein have not yet been elucidated. In the present study, we examined the inflammation-associated signaling which is influenced by brazilein in LPS-induced Raw264.7 macrophage cells.

## 2. Results

### 2.1. Effect of Brazilein on the Inflammatory Products in LPS-Induced Raw264.7

**Figure 1:**
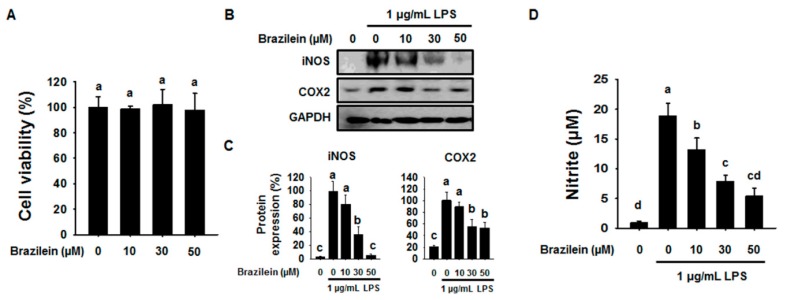
Brazilein attenuated inducible nitric oxide synthase (iNOS) and cyclooxygenase-2 (COX2) protein levels and nitrite production in lipopolysaccharide (LPS)-induced Raw264.7. (**A**) Raw264.7 (5 × 10^3^ cells/well) were incubated with 10, 30 or 50 μM brazilein for 24 h. Cytotoxic evaluation of brazilein on Raw264.7. (*n* = 5); (**B**) Raw264.7 (5 × 10^5^ cells/well) were pre-incubated with 10, 30 or 50 μM brazilein for 1 h and then treated with 1 μg/mL LPS for an additional 24 h. DMSO was used as a vehicle. The protein levels of iNOS and COX2 were determined by western blotting; (**C**) The quantification histogram of iNOS and COX2 protein expression normalized by GAPDH. (*n* = 3); (**D**) Culture media was subsequently isolated and nitrite concentrations determined. The data are expressed as mean ± standard deviations (SD), (*n* = 3). Values with different letters are significantly different, *p* < 0.05.

To determine the cytotoxicity effect of Brazilein on Raw264.7 macrophage cells, we performed XTT assay. According to the results, different concentrations of brazilein had no significant effects on cell cytotoxicity after 24 h incubation. As shown in Figure 1A, brazilein at 10, 30 and 50 μM were found to be non-toxic to Raw264.7. Thus, the concentration of 0, 10, 30 and 50 μM brazilein was selected for the further investigation.

Next, to evaluate the effect of brazilein on inflammation, we analyzed the expression of inflammatory genes or the level of NO by western blotting and cytokine assay. LPS has been known to induce the expression of iNOS and COX2 in Raw264.7 cells and promotes to increase the level of NO. As shown in Figure 1B,C, LPS increased the expression levels of iNOS and COX2 in Raw264.7, while both iNOS and COX2 protein were significantly decreased in Raw264.7 with the presence of brazilein.

Since brazilein led to act as an iNOS suppressor, we also confirmed to NO levels using nitrite colorimetric assay in LPS-stimulated Raw264.7 with the presence or absence of brazilein. We found that brazilein showed significant inhibitory effect on the nitrite production in a dose dependent manner compared to LPS-stimulated Raw264.7 as shown in Figure 1D, suggesting that the concentration of 50 μM brazilein is pharmacologically effective.

### 2.2. Brazilein Attenuates the mRNA Levels of Pro-Inflammatory Cytokines in LPS-Induced Raw264.7

The inflammatory cytokines play important roles in the extent of inflammation and recruit other immune cells that are implicated in the pathogenesis of inflammatory conditions [26]. Brazilein repressed the protein expression levels of COX2 and iNOS from LPS-induced Raw264.7. We next examined the transcription levels of inflammatory cytokine. For this objective, the mRNA levels of inflammatory transcripts were analyzed by semi-quantitative RT-PCR.

**Figure 2:**
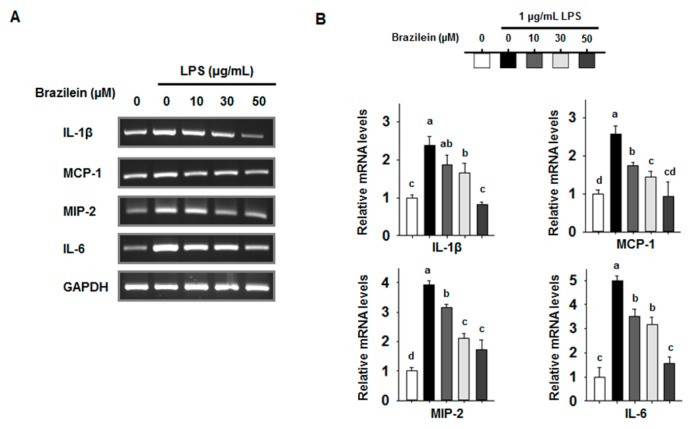
The transcription levels of interleukin (IL)-1β, MCP-1, macrophage inflammatory protein-2 (MIP-2), and IL-6 were decreased by brazilein in LPS stimulated Raw264.7. Cells were pre-incubated with 10, 30 and 50 μM brazilein for 1 h, treated with 1 μg/mL LPS, and incubated for a further 24 h. DMSO was used as a vehicle. (**A**) Total RNA was amplified by RT-PCR using the indicated primers for IL-1β, MCP-1, MIP-2, and IL-6; (**B**) The relative fold changes in mRNA levels were quantified using the ImageJ program. The data are expressed as mean ± standard deviations (SD), (*n* = 3). Values with different letters are significantly different, *p* < 0.05.

**Figure 3:**
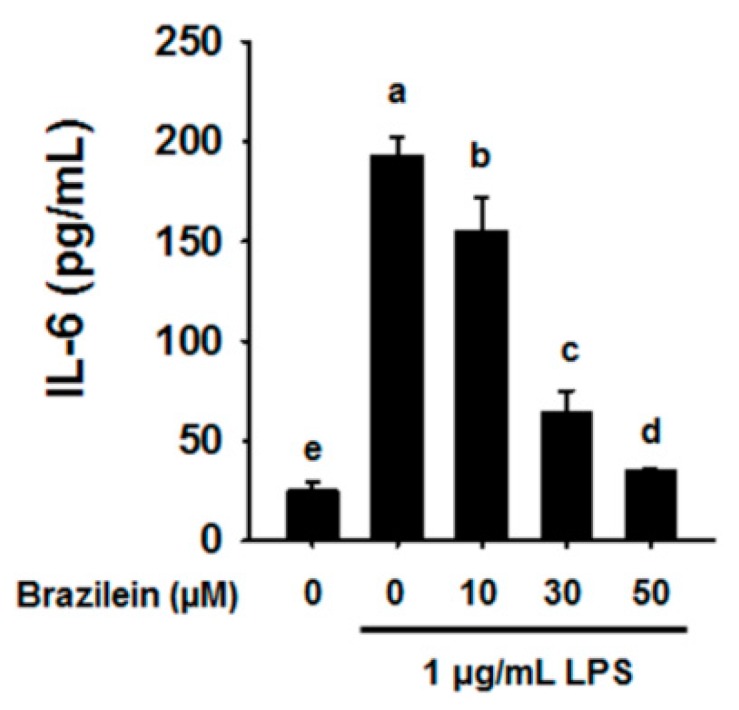
Brazilein suppressed IL-6 cytokine production in LPS-induced Raw264.7. Cells were pretreated with 10, 30, or 50 μM brazilein for 1 h, treated with 1 μg/mL LPS, and incubated for a further 24 h. The production of IL-6 cytokine was quantified using a specific antibody-coated ELISA kit. DMSO was used as a vehicle. The data are expressed as mean ± standard deviations (SD), (*n* = 3). Values with different letters are significantly different, *p* < 0.05.

As shown in Figure 2, brazilein decreased the transcription levels of gene encoding IL-1β, IL-6, MCP-1, and MIP-2 in a dose dependent manner in LPS-treated Raw264.7. However, TNFα level was reduced to a lesser degree by 30 μM brazilein (data not shown). Indeed, brazilein also suppressed the production of IL-6 cytokine in a dose-dependent manner as shown in Figure 3.

### 2.3. Brazilein Decreased NF-κB Luciferase Activity in LPS-Induced Raw264.7

**Figure 4:**
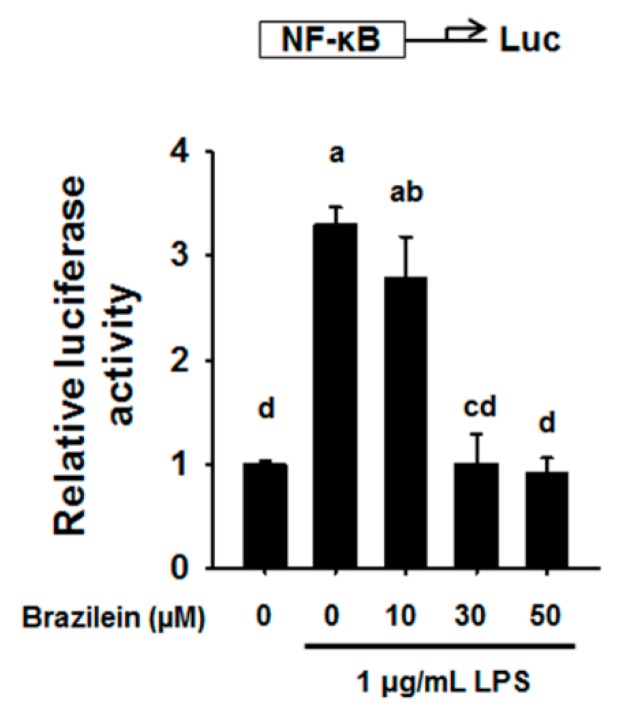
Brazilein attenuated the NF-κB reporter activity in LPS-induced Raw264.7. Cells were pre-incubated with 10, 30, and 50 μM brazilein for 1 h and then co-treated with 1 μg/mL LPS for additional 10 h. DMSO was used as a vehicle. The data are expressed as mean ± standard deviations (SD), (*n* = 3). Values with different letters are significantly different, *p* < 0.05.

NF-κB play a pivotal role in inflammation as a transcription factor of pro-inflammatory cytokines and induce COX2 up-regulation [26]. To measure of transcriptional regulatory activity of NF-κB, luciferase assay has been conducted. Base on luciferase reporter gene assay, it was revealed that the transcriptional regulatory activity of NF-κB was significantly increased in LPS-stimulated Raw264.7, while transcriptional regulatory activity of NF-κB was inhibited by 10, 30 and 50 μM brazilein, suggesting that brazilein regulates the activity of NF-κB as shown in Figure 4.

### 2.4. Brazilein Inhibited the Upstream Target of NF-κB in LPS-Stimulated Raw264.7

**Figure 5:**
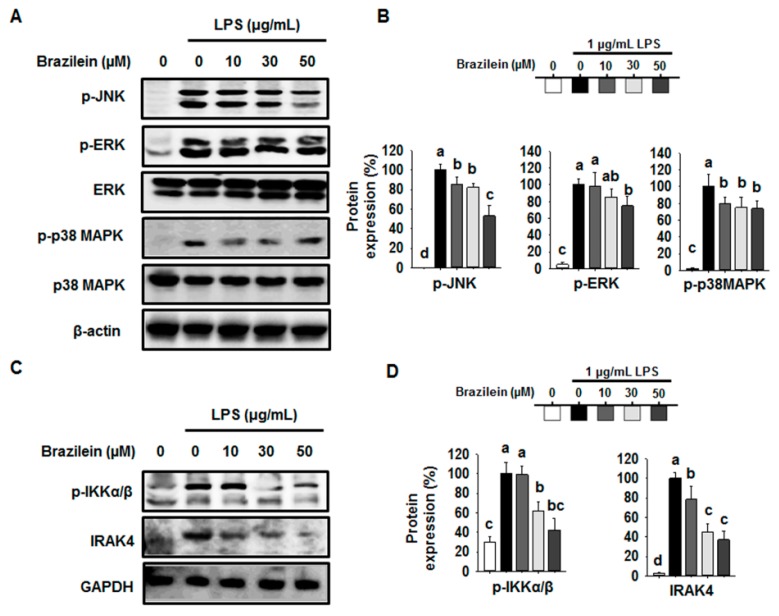
Effect of brazilein on the expression of p-IKKβ, IRAK4, and MAPK signaling pathway in LPS stimulated Raw264.7. Cells were pretreated with 10, 30 or 50 μM brazilein for 1 h and then stimulated with 1 μg/mL LPS for 30 min or 24 h. DMSO was used as a vehicle. Equal amounts of protein in cell lysates were analyzed by Western blot. GAPDH protein levels were used to confirm that equal amounts of protein were subjected to electrophoresis. (**A**) The expression levels of c-Jun terminal kinase (JNK), extracellular signal-regulated protein kinase (ERK)1/2, and p38MAPK protein; (**B**) The quantification histogram of p-JNK, p-ERK1/2, and p-p38MAPK protein expression normalized by β-actin; (**C**) Expression of p-IKKβ and IRAK4 protein; (**D**) The quantification histogram of p-IKKα/β and IRAK4 protein expression normalized by β-actin. The data are expressed as mean ± standard deviations (SD), (*n* = 3). Values with different letters are significantly different, *p* < 0.05.

Next, we examined the effect of brazilein on upstream signaling for NF-κB activation to identify the mechanism of brazilein underlying NF-κB inhibition. First, we determine the phosphorylation of MAPKs pathway, which are intermediate stage controlling of NF-κB activation. As shown in Figure 5A,B, the phosphorylation of JNK, ERK, and p38MAPK were decreased by brazilein, suggesting that the activity of upstream kinases for JNK, ERK, and p38MAPK could also be modulated by brazilein. In fact, IRAK4 is known as the upstream enzymes responsible for phosphorylating MAPK [9].

Next, we determined the expression levels of IRAK4 and p-IKKα/β, which are major mediators controlling the NF-κB activation in LPS-induced inflammatory pathway. Interestingly, brazilein decreased the phosphorylation of IKKα/β at 30 min in LPS-induced Raw264.7 (Figure 5C,D). Since the phosphorylation of IKKα/β is mediated by the activation of IRAK4 kinase, we confirmed the inhibitory activity of brazilein on the expression of IRAK4. We sought that brazilein suppressed the expression of IRAK4 in a dose dependent manner in LPS-induced Raw264.7 compared to the control, indicating that the IRAK4 mediated NF-κB activation pathway might be targeted by brazilein.

## 3. Discussion

Traditional herbal medicine has been used to prevent certain type of diseases worldwide [27,28]. Brazilein is a bioactive compound extracted from *Caesalpinia sappan Linn.* Previously, several studies showed that brazilein exhibited immune suppressive effect and anti-oxidative function [22,24]. However, the molecular targets of brazilein for inflammation prevention have remained elusive. In this study, we evaluated how brazilein regulates the inflammation in LPS-induced Raw264.7, and demonstrated that the brazilein suppresses inflammation through inhibition of the IRAK4-NF-κB pathway.

NF-κB is a transcription factor that promotes the activation of the expression of inflammatory cytokine and its downstream targets such as iNOS. The activation of NF-κB has been shown to be a major signaling pathway involving the suppression of exogenous pathogen mediated inflammation. The NF-κB signaling pathway involves multiple steps including the phosphorylation, ubiquitination, and protein degradation of IKKβ, which leads to NF-κB translocation from cytoplasm to nuclear. A recent study showed that brazilein decreases the nuclear protein expression level of NF-κB [29]. Our study also indicated that brazilein inhibited the iNOS and COX2 expression levels through the down-regulation of IKKβ and the subsequent attenuation of the DNA binding activity of NF-κB.

MAPK pathway is one of the kinase complexes promoting the phosphorylation of NF-κB, which enhances the function of NF-κB in nucleus [30]. NF-κB plays an essential role in the activity and expression of AP-1, which is a nuclear target of JNK signaling; these two factors exhibit mutual positive regulation [31,32]. Indeed, our previous study and others indicated that bioactive compound shows the suppression effect of inflammation through inhibition of JNK, ERK, and p38MAPK mediated NF-κB pathway in macrophage [33,34,35,36]. Similarly, our data also showed that brazilein dramatically decreased the JNK phosphorylation in a dose dependent manner. ERK and p38MAPK showed a tendency to decrease after brazilein treatment, but not in a dose dependent manner. This result indicates that brazilein repressed the JNK phosphorylation and subsequent suppression of the nuclear translocalization of NF-κB in LPS stimulated Raw264.7.

TLRs, receptors that recognize the molecular patterns of pathogens, contribute to a well-coordinated signaling defense system associated with NF-κB activation. IRAK4 has been shown to play a crucial role in TLR mediated signaling. IRAK4 kinase deficient mice have been shown to be resistant to LPS-stimulated inflammation, due to TLR mediated activation of pro-inflammatory cytokine productions [37]. IRAK4 also lead to increase of MAPK signaling pathway, IκB kinase, and NF-κB protein. Then, the activated NF-κB promoted the expression of the downstream target inflammatory cytokines. In this study, we also found that brazilein dramatically suppressed inflammatory cytokines through the regulation of IRAK4 protein level in LPS stimulated Raw264.7.

## 4. Experimental Section

### 4.1. Materials

Lipopolysaccharide (LPS; *Escherichia coli* 0111:B4) was purchased from Sigma (St. Louis, MO, USA). Brazilein was obtained from dried *C. sappan* L. heartwood (purchased in Traditional Medicine Market, Seoul, Korea) methanol extracts, and was purified according to the methods described by Oh *et al.* [38]. The chemical structure of brazilein was identified by the Korea Research Institute of Bioscience & Biotechnology (Sei-Ryang Oh, Ph.D.). Antibodies against phospho-ERK1/2, phospho-JNK, β-actin, and GAPDH were obtained from Santa Cruz Biotechnology (Santa Cruz, CA, USA). Antibodies specific for iNOS, COX2, JNK, p38MAPK, phospho-p38MAPK, ERK1/2, phospho-IKKα/β, and IRAK4 were obtained from Cell Signaling Technology (Danvers, MA, USA). The Dulbecco's modified Eagle medium (DMEM), fetal bovine serum (FBS) and penicillin streptomycin (PS) were purchased from Invitroten (Carlsbad, CA, USA). Unless otherwise noted, all other chemicals were obtained from Sigma (St. Louis, MO, USA).

### 4.2. Cell Line

The murine macrophage cell line, Raw264.7 was purchased from the American Type Culture Collection (Manassa, VA, USA) and maintained in DMEM containing 10% FBS and 1% PS at 37 °C in a 5% CO_2_ incubator.

### 4.3. Evaluation of the Cell Cytotoxicity

Cell cytotoxicity was evaluated by an XTT reduction assay according to the method described by Kim *et al.* [34]. For cell cytotoxic assay, Raw264.7 (5 × 10^3^ cells/well) were cultured in the presence of brazilein in 96-well plates. After 24 h, XTT assay was measured using Colormetric proliferation assay kit (Sigma, Taufkirchen, Germany). XTT reagent was added to the cultures and incubated for 2 h at 37 °C under 5% for 4 h at 37 °C under 5% CO_2_ incubator. Absorbance was measured using an ELISA reader at 570 nm (Bio-Tek Instruments, Winooski, VT, USA).

### 4.4. Transient Transfection and Luciferase Assays

For transfection, Raw264.7 was seeded at a density of 7 × 10^4^ cells/well in 48-well plates and maintained for 24 h until approximately 70%–80% confluence. NF-κB (2×)-luciferase reporter plasmids or the corresponding empty vector plasmids were co-transfected to Raw264.7 using SuperFect Transfection Reagent (Santa Clarita, CA, USA), according to the manufacturer’s instructions. After 24 h transfection, Raw264.7 was treated with the brazilein for 24 h and then lysed in lysis buffer. Luciferase activity was measured using a Wallac Victor2 luminometer (Wlathan, MA, USA).

### 4.5. Nitrite Colorimetric Assay

Raw 264.7 was plated at 7 × 10^4^ cells/well in 96-well plates and incubated for 24 h. Raw264.7 was incubated with brazilein for 1 h and then treated with 1 μg/mL LPS for an additional 24 h. For nitrite determinations, 100 μL of culture supernatant was mixed with same amount of volume of Griess reagent and the absorbance at 540 nm was measured. The NaNO_2_ standard curve was used to determine total nitrite.

### 4.6. RNA Extraction and Semi-Quantitative RT-PCR

RNA was isolated from Raw264.7 using Trizol^®^ reagent (Carlsbad, CA, USA), according to the protocol. Total RNA (1 μg/sample) was incubated with the cDNA synthesis kit (Maxime RT PreMix, Intron Biotechnology, Seongnam, Korea) and incubated at 45 °C for 60 min. The cDNA was then amplified with an Inno Hot Tap polymerase Kit (Bookyung SM, Seoul, Korea). The primer sequences were as follows: IL-1β, forward (5′-CAGGATGAGGACATGAGCACC-3′) and reverse (5′-CTCTGCACACTCAAACTCCAC-3′); MCP-1, forward (5′-GCTGACCCCAAGAAGGAATG-3′) and reverse (5′-GTGCTTGAGGTGGTTGTGGA-3′); MIP-2, forward (5′-GAACAAAGGCAAGGCTAACTGA-3′) and reverse (5′-AACATAACAAVATCTGGGCAAT-3′); IL-6, forward (5′-GTTCTCTGGGAAATCGTGGA-3′) and reverse (5′-TGTACTCCAGGTAGCTA-3′); GAPDH, forward (5′-AACTTTGGCATTGTGGAAGG -3′) and reverse (5′-ACACATTGGGGGTAGGAACA-3′). PCR amplification conditions were: initial denaturation at 94 °C for 3 min; 25–30 cycles of denaturation at 94 °C for 30 s, annealing at 55–57 °C for 30 s, and extension at 72 °C for 1 min; and final extension at 72 °C for 5 min. Amplified products were analyzed on 1% agarose gel containing ethidium bromide. Images were visualized with a Gene Fresh ultraviolet (UV) detector (Syngene, Frederick, MD, USA). Image analysis was conducted using the ImageJ program (National Institutes of Health, Bethesda, MD, USA). The results are representative of three independent experiments.

### 4.7. Enzyme-Linked Immunosorbent Assay (ELISA)

Raw 264.7 was incubated with brazilein for 1 h and then treated with 1 μg/mL LPS for 24 h. The supernatants were collected and stored at −80 °C until use. The level of IL-6 cytokine was determined using ELISA MAX™ Kits (BioLegend, San Diego, CA, USA), following the manufacturer’s instructions.

### 4.8. Western Blotting

Cells were harvested with ice-cold RIPA buffer (50 mM Tris–HCl, 1 mM EDTA, 1 mM EGTA, 150 mM NaCl, 1% NP-40, 0.1% SDS, 0.25% sodium deoxycholate, and 5% β-mercaptoethanol) with protease and phosphatase inhibitors. Cell lysates were centrifuged at 12,000× *g* for 5 min at 4 °C. The protein concentration in the supernatants was determined by the Bradford Protein Assay (Bio-Rad, Richmond, CA, USA). Protein samples (20–30 μg) were separated by SDS-polyacrylamide gel electrophoresis (8%–12%, SDS-PAGE), and transferred onto polyvinylidene fluoride (PVDF) membranes (Bio-Rad, Hercules, CA, USA). The membranes were blocked in a blocking buffer containing 5% non-fat dried milk and were blotted with the indicated primary antibody (1:1000) overnight at 4 °C. Bound primary antibodies were detected with a peroxidase-coupled secondary antibody (1:5000). The reactive bands were visualized by chemiluminescence (Amersham Biosciences, Amersham, UK).

### 4.9. Statistical Analysis

Values are represented as mean ± standard deviation (SD). Statistical analysis was calculated by one-way analysis of variance (ANOVA) with Duncan’s multiple range tests (9.01, SAS program, Raleigh, NC, USA). Values with different letters are significantly different, *p* < 0.05.

## 5. Conclusions

In this study, we demonstrated that the suppression of IRAK4 by brazilein lead to inactivation of MAPK signaling pathway, involving JNK, ERK, and p38 MAPK, and IKKβ, which resulted in inhibition of NF-κB and COX2, and subsequent inactivation of the inflammation end products such as pro-inflammatory cytokines and nitrite. Therefore, we suggest that brazilein might be a useful bioactive compound for the prevention of IRAK4-NF-κB pathway associated chronic diseases.
